# Arginine Supplementation for Juvenile Florida Pompano (*Trachinotus carolinus*) in Practical Plant‐Based Diets

**DOI:** 10.1155/anu/1766363

**Published:** 2026-10-19

**Authors:** Trinh H. V. Ngo, Khanh Q. Nguyen, Timothy J. Bruce, Nicholas Romano, Marty Riche, D. Allen Davis

**Affiliations:** ^1^ School of Fisheries Aquaculture and Aquatic Sciences Auburn University Auburn Alabama USA auburn.edu; ^2^ United States Department of Agriculture Agricultural Research Service Fort Pierce Florida USA usda.gov; ^3^ Harbor Branch Oceanographic Institute Florida Atlantic University Fort Pierce Florida USA fau.edu

**Keywords:** amino acid nutrition, arginine supplementation, gene expression, plant-based diet, *Trachinotus carolinus*

## Abstract

This study aimed to evaluate the effects of dietary arginine (Arg) supplementation on juvenile Florida pompano (*Trachinotus carolinus*). Diet formulation was based on the Arg requirement reported for golden pompano (*Trachinotus ovatus*), 2.73% of diet. Eight isonitrogenous (40% crude protein) and isolipidic (8% crude lipid) diets were formulated with varying levels of L‐arginine supplementation (0%, 0.05%, 0.1%, 0.2%, 0.3%, 0.4%, 0.5%, and 0.65%), resulting in dietary Arg concentrations ranging from 2.32% to 2.94% of the dry diet (5.66% to 6.95% of dietary protein). Five replicate groups of 12 juvenile pompano at an initial weight of 11.06 ± 0.27 g (mean ± standard deviation) were fed these experimental diets for 12 weeks in a recirculating aquaculture system. Growth performance parameters, including final weight (53.06–59.96 g), percentage weight gain (381%–442%), feed conversion ratio (1.64–1.80), thermal‐unit growth coefficient (0.58–0.68), survival (96.67%–100%), and apparent net protein retention (24.48%–26.79%), showed no significant differences among treatments. Whole‐body proximate composition, amino acid profiles, and serum biochemical parameters remained unaffected by different dietary Arg levels. Gene expression analysis of hepatic (*ppara*, *igf1*, *tor*, *mnsod*) and intestinal (*ocln*, *zo1*, *lyz*, *il8*) target genes similarly revealed no significant treatment effects, indicating that hepatic metabolic regulation, intestinal barrier integrity, and mucosal immune status were maintained at the tested Arg range. Under these conditions, results suggest that the dietary Arg requirement of juvenile Florida pompano is ≤2.32% of diet (≤5.66% of dietary protein), and that conventional animal and plant protein ingredients supply adequate Arg to support growth performance, feed efficiency, and normal physiological function. Thus, Arg supplementation may not be required even in low‐animal‐protein diets, supporting more cost‐effective feed formulation.

## 1. Introduction

Reducing feed costs through partial or complete replacement of animal protein with plant‐based protein ingredients is a central challenge in aquaculture diet formulation. In Florida pompano (*Trachinotus carolinus*) aquaculture, numerous plant protein sources, including soybeans, corn, and cotton, have been successfully evaluated and incorporated into diets to partially or entirely replace fish meal (FM) and other animal proteins [1–6]. However, decreasing animal protein sources from 15% to 0% in Florida pompano diets resulted in a consistent decline in growth performance [7, 8]. While plant‐based protein sources can be incorporated into Florida pompano diets, research has demonstrated that supplemental animal protein sources are necessary to support adequate growth and feed utilization [4, 7–10]. This growth depression when animal proteins are removed or restricted is frequently attributed to amino acid (AA) imbalances, antinutritional factors, or the absence of specific nutrients found in animal ingredients. Although considerable progress has been made in lowering animal protein levels, continued research is essential to identify the limiting nutrients in these alternative diet formulations and to determine precise essential amino acid (EAA) requirements for cost‐effective crystalline AA supplementation.

EAAs are often limiting in practical feed formulations, especially in diets with lower animal protein content. Based on established strategies for replacing FM and other animal proteins in Florida pompano diets, taurine, methionine, and lysine have been identified as the primary limiting AAs, and their requirements have been established [11–14]. Furthermore, AA retention analysis suggests that arginine (Arg) may be a limiting EAA in typical low‐FM feed formulations for Florida pompano [15], especially given that the dietary Arg requirement of the closely related golden pompano (*Trachinotus ovatus*) has been established by Lin et al. [16]. Determining whether Florida pompano meets this specific metabolic demand is essential for optimizing crystalline AA supplementation strategies in low‐FM diets and for further reducing the use of costly animal protein sources.

Beyond its role as a substrate for protein synthesis, Arg is classified as a functional AA that influences physiological function and immune status. Arg serves as a precursor for nitric oxide (NO), polyamines, and creatine, thereby influencing vascular regulation, immune response, cellular proliferation, and gastrointestinal integrity [17–19]. Research has demonstrated that plasma‐free Arg levels decline significantly in fish exposed to stress compared with nonstressed individuals [20, 21]. Arg plays an established role in polyamine synthesis, which supports fish growth [17, 22–25], and NO production, which enhances immune response and disease resistance during stress [26, 27] and cellular signaling [28, 29]. Additionally, Arg is a potent stimulator of insulin secretion, exerting stronger insulinotropic effects than glucose, leading to increased circulating concentrations of insulin, IGF‐1, growth hormone, glucagon, and glucagon‐like peptide‐1 [19, 30].

In marine fish species, dietary Arg supplementation beyond requirements for protein synthesis was shown to promote growth [31], improve components of the innate immune system [32–34], and reduce cortisol levels in stressed fish [35]. However, responses are species‐specific. In gilthead seabream (*Sparus aurata*), Arg supplementation did not affect growth, feed utilization, intestinal nutrient absorption capacity, AA metabolism, or oxidative stress [36, 37]. In European seabass (*Dicentrarchus labrax*), Arg supplementation above the growth requirement paradoxically impaired immune status before bacterial challenge, as evidenced by decreased respiratory burst activity of circulating leucocytes and downregulated expression of immune‐related genes in the spleen and intestine and subsequently increased susceptibility to vibriosis upon intracoelomical challenge with *Vibrio anguillarum* [38]. In juvenile golden pompano, growth performance and protein utilization efficiency peaked at the estimated dietary Arg requirement of 2.73% dry diet and declined significantly when dietary Arg exceeded this level [16]. This decline may reflect antagonism with lysine, impairing its intestinal absorption [39, 40] or increase ureagenesis at high Arg levels [37, 41].

Despite the physiological importance of Arg, the economic need for precise feed formulation in Florida pompano aquaculture, and the species‐specific variability observed in related marine fishes, the exact dietary Arg requirement for juvenile Florida pompano remains unknown. Given their taxonomic proximity to golden pompano and the critical role of Arg in both growth and immune function, estimating requirements from related species may lead to inefficient feed formulations. Therefore, this study aimed to evaluate the effects of graded dietary Arg supplementation on growth performance, feed utilization, whole‐body composition, serum biochemistry, and hepatic and intestinal gene expression in juvenile Florida pompano (*Trachinotus carolinus*) through a dose–response approach.

## 2. Materials and Methods

### 2.1. Experimental Diets

Eight isonitrogenous (40% crude protein) and isolipidic (8% lipid) diets with graded Arg levels (2.32%–2.94% of diet, as‐is basis) were formulated to evaluate the effects of Arg supplementation on juvenile Florida pompano (*Trachinotus carolinus*). All experimental diets contained a 4% Arg‐free AA premix reflecting pompano whole‐body AA composition [15], which was incorporated into all diets (Table 1). Crystalline L‐arginine supplementation at increasing levels, with corresponding inverse adjustments in glycine, produced the arginine gradient while maintaining balanced AA profiles. All diets were prepared at the Aquatic Animal Nutrition Laboratory, School of Fisheries, Aquaculture, and Aquatic Sciences (SFAAS) at Auburn University (Auburn, Alabama, USA), following established procedures for preparing experimental fish feeds [15]. Dry ingredients were ground and weighed and then combined with oil and homogenized in a Hobart food mixer (Troy, Ohio, USA) for 15 min. Boiling water was gradually added to form a dough‐like consistency suitable for pelleting. Diets were pressure‐pelleted through a 3 mm die using a meat grinder and then dried in a forced‐air oven at ~35°C until the moisture content was reduced below 10%. The dried diets were sealed in plastic bags and stored at −20°C before use in the feeding trial. Prior to feeding, diets were ground and sieved to achieve particle sizes appropriate for the experimental fish sizes. Proximate composition and AA profiles of all diets (Table 2) were determined from a single composite sample per diet representing the manufacturing batch, analyzed by the University of Missouri Agricultural Experiment Station Chemical Laboratories (Columbia, Missouri, USA).

**Table 1:** Ingredient composition of experimental diets (g 100 g ^−1^, as is) based on isonitrogenous and isolipidic (40 g 100 g^−1^ protein and 8 g 100 g^−1^ lipid) basis, with various arginine levels used to assess Florida pompano (*T*. *carolinus*) cultured in a clear water recirculating system for 12 weeks.

Ingredients	Basal	A005	A01	A02	A03	A04	A05	A065
Poultry meal^a^	14.00	14.00	14.00	14.00	14.00	14.00	14.00	14.00
Soybean meal^b^	44.00	44.00	44.00	44.00	44.00	44.00	44.00	44.00
Corn protein concentrate^c^	6.50	6.50	6.50	6.50	6.50	6.50	6.50	6.50
Fish oil^d^	3.80	3.80	3.80	3.80	3.80	3.80	3.80	3.80
Soy oil	1.77	1.77	1.77	1.77	1.77	1.77	1.77	1.77
Lecithin^e^	0.50	0.50	0.50	0.50	0.50	0.50	0.50	0.50
Corn starch^f^	9.38	9.38	9.38	9.38	9.38	9.38	9.38	9.38
Whole wheat^g^	12.00	12.00	12.00	12.00	12.00	12.00	12.00	12.00
Mineral premix^h^	0.25	0.25	0.25	0.25	0.25	0.25	0.25	0.25
Vitamin premix^i^	0.50	0.50	0.50	0.50	0.50	0.50	0.50	0.50
Choline chloride^j^	0.20	0.20	0.20	0.20	0.20	0.20	0.20	0.20
Stay‐C 35%^k^	0.10	0.10	0.10	0.10	0.10	0.10	0.10	0.10
CaP‐dibasic^l^	1.75	1.75	1.75	1.75	1.75	1.75	1.75	1.75
Arginine^m^	0.00	0.05	0.10	0.20	0.30	0.40	0.50	0.65
DL‐methionine^n^	0.10	0.10	0.10	0.10	0.10	0.10	0.10	0.10
Glycine^n^	0.65	0.60	0.55	0.45	0.35	0.25	0.15	0.00
Taurine^n^	0.50	0.50	0.50	0.50	0.50	0.50	0.50	0.50
AA premix^o^	4.00	4.00	4.00	4.00	4.00	4.00	4.00	4.00

^a^River Valley Ingredients, Hanceville, Alabama, USA.

^b^De‐hulled solvent‐extracted soybean meal, Bunge Limited, Chesterfield, Missouri, USA.

^c^Empyreal 75, Cargill Corn Milling, Cargill Inc., Blair, Nebraska, USA.

^d^Omega Protein Inc., Houston, Texas, USA.

^e^The Solae Company, St. Louis, Missouri, USA.

^f^Ingredi Company, 501 Chesapeake Park Plaza, Baltimore, Maryland, USA.

^g^Archer Daniels Midland, 4666 E Faries Pkwy, Decatur, Illinois, USA.

^h^Mineral premix (g 100 g^−1^ premix): cobalt chloride, 0.008; cupric sulfate pentahydrate, 0.01; ferrous sulfate heptahydrate, 20; manganous sulfate anhydrous, 2.90; potassium iodide, 0.240; sodium selenite, 0.048; zinc sulfate heptahydrate, 17.600 and α cellulose 59.194.

^i^Vitamin premix (g 100 g^−1^ premix): thiamin HCL, 8.0; riboflavin, 8.0; pyridoxine HCl, 5.0; Ca‐pantothenate, 20.0; niacin, 40.0; biotin, 0.040; folic acid, 1.80; cyanocobalamin, 0.002; vitamin A acetate (500,000 IU g^−1^), 2.40; vitamin D3 (400,000 IU g^−1^), 0.50; DL‐α‐tocopheryl acetate, 80.0; and α cellulose, 834.258.

^j^MP Biomedicals Inc., Solon, Ohio, USA.

^k^Stay *C*, (L‐ascorbyl‐2‐polyphosphate 35% Active C), Roche Vitamins Inc., Parsippany, New Jersey, USA.

^l^Bean Town Chemical, 9 Sagamore Park Road, Hudson, New Hampshire, USA.

^m^VWR Chemicals, LLC, Radnor, Pennsylvania, USA.

^n^Tokyo Chemical Industry, Portland, Oregon, USA.

^o^Amino acid premix (g 100 g^−1^): Thr 0.532; Val 0.604; DL‐Met 0.354; Ile 0.529; Leu 0.877; Phe 0.501; Lys 1.00; Asp 1.153; Glu 1.687; Pro 0.632; Ala 0.840 and Tyr 0.388.

**Table 2:** Amino acid profile (g 100 g^−1^ as is) of each diet based on isonitrogenous and isolipidic (40 g 100 g^−1^ protein and 8 g 100 g^−1^ lipid) basis, with various arginine levels used to assess Florida pompano (*T*. *carolinus*) cultured in a clear water recirculating system for 12 weeks.

Amino acids (AAs) ^∗^	Basal	A005	A01	A02	A03	A04	A05	A065
Essential AAs								
Arginine	2.32	2.38	2.43	2.52	2.62	2.76	2.81	2.94
Histidine	0.86	0.85	0.89	0.88	0.88	0.90	0.89	0.89
Isoleucine	1.84	1.76	1.85	1.84	2.14	1.85	1.81	1.82
Leucine	3.49	3.46	3.59	3.58	3.55	3.61	3.64	3.60
Lysine	2.40	2.39	2.49	2.45	2.46	2.53	2.48	2.48
Methionine	0.85	0.83	0.88	0.85	0.83	0.89	0.88	0.88
Phenylalanine	1.99	2.05	2.10	2.06	2.04	2.10	2.08	2.08
Threonine	1.51	1.55	1.56	1.58	1.55	1.64	1.61	1.65
Tryptophan	0.34	0.37	0.37	0.35	0.36	0.37	0.36	0.36
Valine	2.03	1.93	2.04	2.01	2.05	2.07	2.05	2.10
Nonessential AAs								
Alanine	2.34	2.37	2.39	2.45	2.42	2.48	2.45	2.50
Aspartic acid	3.80	3.84	3.92	3.91	3.87	4.06	3.97	4.03
Cysteine	0.52	0.49	0.53	0.52	0.52	0.55	0.55	0.55
Glutamic acid	7.23	7.20	7.54	7.45	7.40	7.58	7.52	7.52
Glycine	2.66	2.57	2.63	2.48	2.32	2.32	2.16	2.15
Hydroxylysine	0.12	0.13	0.12	0.12	0.12	0.12	0.12	0.13
Hydroxyproline	0.40	0.42	0.39	0.37	0.39	0.40	0.38	0.45
Ornithine	0.03	0.03	0.03	0.03	0.03	0.03	0.03	0.03
Proline	2.55	2.55	2.57	2.58	2.60	2.62	2.60	2.66
Serine	1.55	1.53	1.58	1.59	1.59	1.64	1.62	1.63
Taurine	0.66	0.67	0.67	0.66	0.65	0.68	0.67	0.67
Tyrosine	1.50	1.46	1.53	1.51	1.50	1.54	1.54	1.54
Total AA	40.99	40.83	42.10	41.79	41.89	42.74	42.22	42.66
Proximate analysis ^∗^								
Crude protein	40.98	41.41	40.99	41.24	41.96	41.78	41.83	42.30
Moisture	8.42	6.46	8.33	7.47	7.71	7.06	6.74	6.03
Crude fat	8.74	8.85	8.56	8.70	8.81	8.73	8.80	8.99
Crude fiber	2.55	2.80	2.55	2.68	2.35	2.40	2.47	2.79
Ash	6.31	6.39	6.47	6.25	6.43	6.52	6.46	6.36
Arginine (% protein)	5.66	5.81	5.87	6.11	6.24	6.61	6.72	6.95

^∗^Values are based on a single composite sample per diet analyzed at the University of Missouri Agricultural Experiment Station Chemical Laboratories (Columbia, Missouri, USA).

### 2.2. Experimental Fish and Growth Trial

Florida pompano fingerlings (~4 g mean weight) were sourced from Florida Atlantic University at Harbor Branch Oceanographic Institute (Fort Pierce, Florida, USA). The fish were placed into a hauling tank with a supplemental oxygen system and transported to the E. W. Shell Fisheries Center in Auburn, Alabama, USA. Upon arrival, the fish were transferred and acclimated to a nursery tank and fed to apparent satiation with a 55% crude protein and 15% crude fat commercial feed (Finfish Starter, Ziegler Bros., Inc., Gardner, Pennsylvania, USA) until they reached an appropriate size of about 10–11 g for the growth trial. At the start of the trial, 12 fish with a mean weight of 11.06 ± 0.27 g (weight ± standard deviation) were stocked into a semiclosed recirculating system consisting of 40 tanks (~135 L capacity, ~0.27 m^2^ bottom surface area) with 100 L working volume, operated at a flow rate of 4.48 L min^−1^ for a turnover time of 2 min, with 5 replicates per dietary treatment. The recirculating system included a central reservoir, Aquadyne bead filters (0.2 m^2^ media, 0.6 m × 1.1 m dimensions), one 0.25 hp centrifugal pump for the filtration system and one 0.25 hp centrifugal pump (RK2, San Marcos, California, USA) for recirculation, and a 50 µm cartridge filter (Pentair plc, Golden Valley, Minnesota, USA) for removing fine particulates. Salinity was maintained at ~14–15 g L^−1^ in the trial using Instant Ocean Sea Salt (Instant Ocean Spectrum Brands, Blacksburg, Virginia, USA). Photoperiod was maintained on a 12 h light and 12 h dark cycle using an automatic indoor lighting system. Fish were fed six times daily at 2%–8% of their body weight. Feeding rates were adjusted weekly based on growth and feeding observations, with amounts recalculated every 2 weeks after sampling to reflect changes in biomass and mortalities. Each tank was cleaned every 2 weeks, and during cleaning, the fish were removed and weighed together. To reduce the risk of parasite infection, fish were prophylactically treated during handling with a 30‐s immersion in formalin (250 mg L^−1^; Parasite‐S, Syndel, Ferndale, WA, USA), followed by a 1 min treatment with chloroquine phosphate (60 mg L^−1^; MP Biomedicals, Solon, Ohio, USA). This treatment was repeated at every handling during the trial.

Water quality parameters were monitored regularly throughout the trial. Dissolved oxygen, temperature, and salinity were measured twice daily at 0730‐0745 and 1530‐1545 using a YSI Pro Plus Meter (YSI Inc., Yellow Springs, Ohio, USA). pH was assessed twice weekly with the pHTestr30 (Oakton Instrument, Vernon Hills, Illinois, USA), while total ammonia nitrogen (TAN) and nitrite were quantified using a YSI EcoSense 9500 Photometer (YSI Inc., Yellow Springs, Ohio, USA). System maintenance procedures included twice‐weekly bead filter backwashing with 20% water exchange, performed on days when TAN and nitrite were measured. Sodium bicarbonate (NaHCO_3_; Feed Products & Service Co., St. Louis, Missouri, USA) was added as necessary to maintain pH and alkalinity.

At the end of the feeding trial, pompano were fasted overnight before sampling, then group‐weighed from each tank, and counted to determine the final biomass, number of fish, and average final weight (FW). Growth and feed utilization metrics, including feed conversion ratio (FCR), percentage weight gain (WG), thermal‐unit growth coefficient (TGC), apparent net protein retention (ANPR), and survival, were calculated. Four fish per tank were randomly selected for whole body and AA proximate analyses. Another four fish were anesthetized with tricaine methanesulfonate (MS‐222; 100 mg L^−1^) for ~2–3 min or until they stopped swimming, and then blood samples were immediately collected from the caudal vein using a 1 mL syringe with a 25‐gauge needle (Exelint International, Redondo Beach, California, USA). These samples were placed in 1.5 mL microcentrifuge tubes without anticoagulant for hematocrit measurement and serum biochemical analysis. Small pieces of liver and distal intestine (~1 cm segment taken 1 cm from the vent) from three euthanized fish per treatment were collected and placed into separate 1.5 mL tubes with RNA Shield (Zymo Research Inc., California, USA) immediately after dissection to preserve RNA. The samples were refrigerated overnight and then stored at −80°C until processed for total RNA extraction and quantitative real‐time PCR (qPCR).

The parameters were calculated as per the following formulas:

Final weight FW;g=Total biomass gFinal fish number,

Percentage weight gain WG; %=Final weight g−Initial weight gInitial weight g× 100,

Survival % =Final fish numberInitial fish number× 100,

Feed conversion ratio FCR=Feed intake FI,dry weight g Weight gain wet weight g,

Thermal-unit growth coefficient TGC=Final weight13/−Initial weight13/Water temperature °C×Days× 100,

Apparent net protein retention ANPR; %=Protein gained g Protein fed g× 100.

### 2.3. Whole‐Body Composition

Upon termination of the trial, 10 fish from the initial population and 4 fish from each replicate were randomly selected and stored at −20°C for whole‐body proximate and AA profile analyses. Before analysis, samples from each tank were thawed, chopped, and homogenized using a food processor to produce a uniform composite sample. Proximate composition and AA profiles were conducted by an independent laboratory.

### 2.4. Blood Biochemistry

Blood samples were collected for hematocrit and serum biochemical analyses. For hematocrit measurement, whole blood was drawn into soda‐lime glass microhematocrit capillary tubes (DWK Life Sciences LLC, Millville, New Jersey, USA), sealed with wax (Paul Marienfeld GmbH & Co. KG, Lauda‐Königshofen, Germany), and centrifuged for 5 min using an IEC Clinical Centrifuge (International Equipment Co., Needham Heights, Massachusetts, USA). Hematocrit values were read with a microcapillary reader (International Equipment Co., Needham Heights, Massachusetts, USA). For serum biochemistry, blood samples were allowed to clot for 4 h at 4°C and then centrifuged at 4000× *g* for 10 min to separate the serum. Three serum samples from each tank were pooled into one 450 μL composite sample per tank in sterile 1.5 mL microcentrifuge tubes and stored at −80°C until the analysis. Serum biochemical parameters were analyzed by WellFish Tech (Charlottetown, Prince Edward Island, Canada).

### 2.5. Gene Expression Analysis

Samples of the distal intestine and liver were transferred into a 96‐deep well plate containing 0.1 mm ceramic beads (Omni International, Kennesaw, Georgia, USA) and homogenized using Bead Ruptor 96 (Omni International, Kennesaw, Georgia, USA) for a total of 20 min at 30 Hz, alternating between 5 min of homogenization and 5 min of rest. Total RNA was extracted and purified from 100 µL of homogenate using the MagMAX mirVana Total RNA Isolation Kit (Thermo Fisher Scientific, Waltham, Massachusetts, USA). RNA concentration and purity were assessed using a BioTek Take3 Trio microvolume spectrophotometer (Agilent, Santa Clara, CA, USA). Mean A260/280 ratios were 2.13 ± 0.02 and 2.10 ± 0.02 for intestine and liver, respectively, and mean A260/230 ratios were 2.38 ± 0.04 and 1.66 ± 0.19 for intestine and liver, respectively. RNA samples were diluted to a uniform concentration of 20 ng μL^−1^ prior to cDNA synthesis.

Complementary DNA was synthesized using the iScript cDNA Synthesis Kit (Bio‐Rad Laboratories, Hercules, California, USA). Each 20 μL reaction contained 4 μL of iScript Reaction Mix, 1 μL of iScript Reverse Transcriptase, 10 μL of template RNA (20 ng μL^−1^), and 5 μL of nuclease‐free water, yielding a cDNA stock concentration of 10 ng μL^−1^. Thermal cycling was performed on a Bio‐Rad T100 thermal cycler (Bio‐Rad Inc., Hercules, California, USA) with the program set at 25°C for 5 min, 46°C for 20 min, and 95°C for 1 min. The cDNA stock was finally diluted to 1.25 ng μL^−1^ with nuclease‐free water and stored at −80°C until the analysis.

Gene expression was quantified by real‐time quantitative PCR using the SsoAdvanced Universal SYBR Green Supermix (Bio‐Rad, Hercules, California, USA) on a Bio‐Rad CFX Opus 384 Real‐Time PCR System. Reactions were performed in Hard‐Shell 384‐well standard PCR plates with white wells (Bio‐Rad, Hercules, California, USA) and sealed using Microseal “B” adhesive optical seals (Bio‐Rad, Hercules, California, USA). Four target genes were quantified in intestinal tissue, including occludin (*ocln*), zonula occludens‐1 (*zo1*), lysozyme (*lyz*), and interleukin‐8 (*il8*), selected to reflect intestinal barrier integrity and mucosal immune status. Four target genes were quantified in liver tissue, including peroxisome proliferator‐activated receptor alpha (*ppara*), insulin‐like growth factor 1 (*igf1*), target of rapamycin (*tor*), and manganese superoxide dismutase (*mnsod*), selected to reflect lipid metabolism, growth axis regulation, protein synthesis signaling, and hepatic antioxidant defense, respectively. Primers for intestinal target and reference genes were sourced from previously published studies in golden pompano [42–44], while primers for hepatic target genes were designed de novo using the National Library of Medicine (NCBI) Primer‐BLAST [45] with *Trachinotus* spp. RefSeq mRNA sequences as design templates, selected as surrogates for *T. carolinus* based on close phylogenetic relationship within the genus and the availability of annotated RefSeq sequences. Primer pairs were designed to span exon–exon junctions to prevent amplification of genomic DNA, with product size constrained to 100–200 bp, primer length optimized at 20 bp (range 18–22 bp), melting temperature optimized at 59°C (range 58–62°C) with a maximum interprimer difference of 1°C, GC content constrained to 40%–60%, and self‐complementarity and 3^′^ self‐complementarity thresholds set to eight and three, respectively. All primer sequences, amplicon lengths, and efficiencies are presented in Table 3.

**Table 3:** Primer sequences, amplicon size, PCR amplification efficiencies, and GenBank accession numbers for target and reference genes used in quantitative RT‐PCR analysis of intestinal and liver gene expression in juvenile Florida pompano (*Trachinotus carolinus*).

Gene	Primer sequence (5^′^→ 3^′^)	Primer efficiency (%)	Amplicon size (bp)	GenBank number	References
Reference genes					
* β-actin*	CGTGCGTGACATCAAGGAGAA AAGGAAGGAAGGCTGGAAGAGG	97.63	178	MK250485	[42]
* 18s*	AGTTGGCATCGTTTATGGTCG GCATTCGTATTGTGCCGCTA	96.16	160	XR_011689216	[42]
* ef1*α	GTCCGTCAAGGAAATCCGTCG TTGAACTTGCAGGCAATGTGAG	105.12	174	XM_071499965	[42]
* b2m*	AAGTCAGTCCACCCAAGGTTCA GGGATTTCCATTCCGTTCTTCATG	107.68	139	XM_071502043	[42]
Intestine target genes					
* ocln*	TACGCCTACAAGACCCGCA CACCGCTCTCTCTGATAAA	105.83	174	XM_071492848	[43]
* zo1*	CGACAAAGAGAAAGGAGAAACG AGAAGTGGTCAATGAGCACAGATA	103.86	191	XM_071496212	[43]
* lyz*	TATGGGCTGATGCAGATTGAC TTTTGCCTTATGCGTGTGATG	96.78	131	KT935523	[44]
* il8*	TGCATCACCACGGTGAAAAA GCATCAGGGTCCAGACAAATC	109.73	140	XM_071503985	[43]
Liver target genes					
* ppara*	AGTGACCTGGCTCTGTTTG AAGCGTCGTCTGGATGATT	108.44	145	XM_071493288	[45]
* igf1*	CGCAATGGAACAAAGTCGG AGGAGATACAGCACATCGCACT	101.37	198	XM_071502597	[45]
* tor*	CGGCTGGATACGACCATACT CGGATGTGCTGATAACCTGC	102.83	148	XM_071463003	[45]
* mnsod*	GACAAGGAGAGTGGAAGGCT GGTAGTAGGCATGCTCCCAT	102.40	115	XM_071479416	[45]

Prior to qPCR assays, primer efficiency for each primer pair was evaluated at an annealing temperature of 58°C, a primer concentration of 500 nM, and a serial dilution of the template cDNA (1:10 dilution series). Primer efficiency was calculated as:

Efficiency % =10−1Slope−1×100.

Only primer pairs with efficiencies between 90% and 110% and a coefficient of determination (*R* ^2^) ≥ 0.99 were accepted for subsequent analyses. Each 10 μL qPCR reaction consisted of 5 μL of SsoAdvanced Universal SYBR Green Supermix (Bio‐Rad, Hercules, California, USA), 0.5 μL each of forward and reverse primer (10 μM stock), and 4 μL of diluted cDNA (1.25 ng μL^−1^). Reactions were performed in duplicate alongside a negative control in which nuclease‐free water replaced the cDNA template. Thermal cycling consisted of an initial incubation at 50°C for 2 min and 95°C for 2 min, followed by 40 cycles of 95°C for 15 s, 58°C for 15 s, and 72°C for 30 s. Melt curve analysis was performed postamplification to confirm product specificity for each primer pair.

Expression stability of four candidate reference genes, *β-actin*, elongation factor 1‐alpha (*ef1a*), β2‐microglobulin (*b2m*), and 18S ribosomal RNA (18 s), was evaluated in both intestine and liver tissues using raw Cq values from all experimental samples as input. Stability was assessed using three complementary algorithms following their original methods. geNorm [46] was used to rank individual gene stability and determine the minimum number of reference genes required for reliable normalization. BestKeeper [47] ranked genes by their correlation with the geometric mean index of stable candidates, with genes exceeding a standard deviation of 1 Ct excluded from index construction. Pairwise gene–gene correlations were additionally computed to screen candidate pairs for potential coexpression, with false discovery rate correction applied to all *p*‐values. NormFinder [48] ranked individual genes while accounting for treatment group structure and identified the most stable reference gene pair by jointly minimizing combined intragroup and intergroup stability values. Where the recommended pair was flagged as coexpressed by BestKeeper, defined as an absolute Pearson *r* ≥ 0.5 with a false discovery rate‐adjusted *p*‐value below 0.05, individual NormFinder stability values were additionally examined to distinguish constitutive from treatment‐driven coregulation. Stability metrics from all three algorithms are presented in Table 4.

**Table 4:** Reference gene stability metrics in intestine and liver of juvenile Florida pompano (*Trachinotus carolinus*).

Tissue	Gene	geNorm M	geNorm rank	NormFinder stability	NormFinder rank	BestKeeper correlation coefficient	BestKeeper rank	Composite geomean	Composite rank
Intestine	*18 s*	0.63	3	0.09	1	0.97	1	1.44	2
	*β-actin*	0.63	1	0.09	1	0.97	2	1.26	1
	*ef1a*	0.63	2	0.11	3	0.96	3	2.62	3
	*b2m*	1.31	4	0.60	4	0.46	4	4.00	4
Liver	*18 s*	0.43	2	0.11	2	0.88	2	2.00	2
	*β-actin*	0.39	1	0.08	1	0.88	1	1.00	1
	*ef1a*	0.46	3	0.13	3	0.84	3	3.00	3
	*b2m*	0.58	4	0.22	4	0.78	4	2.00	4

*β-Actin* and *18S* rRNA were identified as the most stable reference gene pair across both intestine and liver tissues, with a NormFinder joint stability score of 0.07, indicating minimal combined within‐ and between‐group variance across dietary Arg treatments. Relative gene expression levels of all target genes were quantified using the 2^−ΔΔCt^ method [46, 49], with the geometric mean Ct of *β-actin* and *18S* rRNA as the normalization factor. Fish fed the basal diet served as the calibrator group, against which all treatment groups were expressed as fold‐change with a relative expression value of 1.

### 2.6. Statistical Analysis

Prior to conducting all analyses, data were examined for normality and homogeneity of variances utilizing the Shapiro–Wilk and Bartlett’s tests, respectively [50, 51], to confirm compliance with the assumptions of parametric analysis. The influence of dietary treatments on growth parameters, whole‐body proximate composition, AA profiles, serum biochemistry, and gene expression was assessed using one‐way analysis of variance (ANOVA), with significant effects followed by Tukey’s Honest Significant Difference test for pairwise comparisons. Statistical significance was set at α = 0.05 for all analyses.

Linear, quadratic, and broken‐line regression models were evaluated for all parameters using the coefficient of determination (*R* ^2^), Akaike information criterion corrected (AICc), weighted AICc, relative likelihood, and evidence ratio [52]. The best‐fit model for each parameter including FW, WG, TGC, and ANPR was selected based on the lowest AICc and highest weighted AICc, and one‐way ANOVA results are reported alongside the *R* ^2^ and *p*‐value of the corresponding best‐fit model. Fitted dose–response curves are presented with 95% confidence intervals.

All statistical analyses and visualizations were conducted in R v4.5.2 [53] and RStudio v2026.1.0 [54], utilizing agricolae v1.3.7 [55], Biobase v2.72.0 [56], boot v1.3.32 [57], car v3.1.5 [58], emmeans v2.0.1 [59], extrafont v0.20.0 [60], factoextra v1.0.7 [61], ggtext v0.1.2 [62], flextable v0.9.10 [63], gtsummary v2.5.0 [64], janitor v2.2.1 [65], minpack.lm v1.2.4 [66], multcomp v1.4.29 [67], MuMIn v1.48.11 [68], NormqPCR v1.58.0 [69], officer v0.7.3 [70], parallel v4.5.2 [53], patchwork v1.3.2 [71], ReadqPCR v1.58.0 [69], scales v1.4.0 [72], segmented v2.2.1 [73], showtext v0.9.7 [74], and tidyverse [75] packages.

## 3. Results

### 3.1. Growth Performance

Water quality remained within acceptable limits for Florida pompano throughout the feeding trial, with DO at 6.67 ± 0.46 mg L^−1^, salinity at 14.72 ± 0.53 g L^−1^, temperature at 28.64 ± 0.59°C, pH at 8.10 ± 0.09, TAN level of 0.26 ± 0.13 mg L^−1^, and nitrite level at 0.04 ± 0.02 mg L^−1^ over the trial period.

Growth performance and feed utilization of juvenile Florida pompano fed with different dietary Arg levels are presented in Table 5. No growth performance index significantly differed among Arg treatments at any level up to 2.94% of dietary after 12 weeks. FW ranged from 53.06 to 59.96 g (*p* = 0.273), percentage WG from 381% to 442% (*p* = 0.309), and the TGC from 0.58 to 0.68 (*p* = 0.283). Sharing a similar outcome, FCR (1.64 to 1.80; *p* = 0.211), ANPR (24.48%–26.79%; *p* = 0.522), and survival (96.67%–100%; *p* = 0.460) did not statistically differ among dietary treatments. Linear, quadratic, and broken‐line models fitted to FW, WG, the TGC, and ANPR produced no significant dose response (*R* ^2^ < 0.03 and *p* > 0.05; Figure 1A–D), and no model resolved a requirement breakpoint within the tested range.

**Figure 1:**
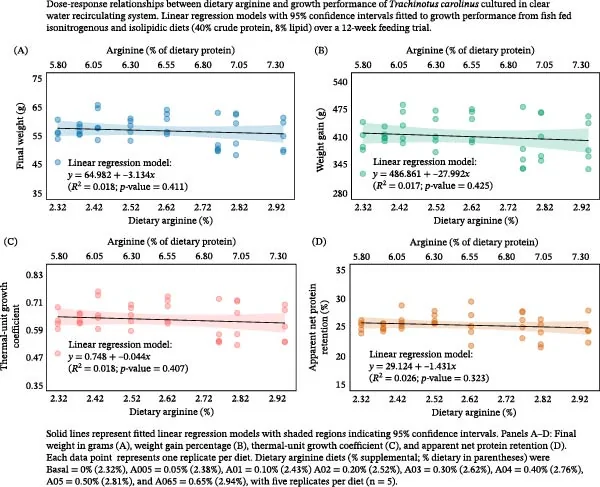
Relationship between dietary arginine and performance metrics of Florida pompano (*Trachinotus carolinus*) fed an isonitrogenous and isolipidic (40% crude protein and 8% lipid) diet for 12 weeks, stocked at 12 fish tank^−1^ (~44 fish m^−2^) with an initial weight of 11.06 ± 0.27 g (mean ± standard deviation). Panels show: (A) final weight (g), (B) weight gain (%), (C) thermal‐unit growth coefficient, and (D) apparent net protein retention (%). Regression curves were fitted using a dose–response model, with 95% confidence intervals.

**Table 5:** Growth performance and feed utilization of Florida pompano cultured in a clear water recirculating system for 12 weeks, fed different arginine levels, stocked at 12 fish tank^−1^ (~44 fish m^−2^) with an initial weight of 11.06 ± 0.27 g (mean ± standard deviation).

Diets	Final weight (g)	Weight gain (%)	FCR	TGC	Survival (%)	ANPR (%)
Basal	54.56	393	1.78	0.60	98.33	25.21
A005	57.05	416	1.68	0.64	98.33	25.04
A01	59.91	442	1.64	0.68	96.67	26.79
A02	57.96	425	1.68	0.65	100.00	26.19
A03	59.96	442	1.65	0.68	100.00	25.59
A04	53.06	381	1.80	0.58	100.00	24.52
A05	57.09	416	1.67	0.64	100.00	24.48
A065	55.14	398	1.70	0.61	98.33	25.35
*p*‐value	0.273	0.309	0.211	0.283	0.460	0.522
PSE	2.15	20	0.05	0.03	1.25	0.82

*Note*: Values represent the mean of five replicates of each diet. Basal = 0% (2.32%), A005 = 0.05% (2.38%), A01 = 0.10% (2.43%), A02 = 0.20% (2.52%), A03 = 0.30% (2.62%), A04 = 0.40% (2.76%), A05 = 0.50% (2.81%), and A065 = 0.65% (2.94%) supplemented arginine (% dietary arginine).

Abbreviations: ANPR, apparent net protein retention; FCR, feed conversion ratio; PSE, pooled standard error; TGC, thermal‐unit growth coefficient.

### 3.2. Whole‐Body Composition

Whole‐body proximate composition and AA profiles were unaffected by dietary Arg level (*p* > 0.05; Table 6). The proximate composition of Florida pompano, including moisture, crude protein, crude fat, crude fiber, and ash, remained consistent across all dietary treatments. Similarly, the profiles of both essential and nonessential AA (on an as‐is basis) showed little variation among the different diets.

**Table 6:** Whole‐body composition and amino acid profiles (g 100 g^−1^, as is) of Florida pompano cultured in a clear water recirculating system for 12 weeks, fed different arginine levels, stocked at 12 fish tank^−1^ (~44 fish m^−2^) with an initial weight of 11.06 ± 0.27 g (mean ± standard deviation).

Amino acids (AAs) ^∗^	Basal	A005	A01	A02	A03	A04	A05	A065	PSE	*p*‐Value
Essential AAs										
Arginine	1.02	1.03	1.05	1.05	1.01	1.07	1.03	1.03	0.029	0.819
Histidine	0.33	0.35	0.34	0.34	0.34	0.35	0.34	0.35	0.011	0.932
Isoleucine	0.61	0.65	0.64	0.63	0.64	0.63	0.60	0.64	0.021	0.833
Leucine	1.05	1.09	1.09	1.07	1.08	1.07	1.04	1.09	0.033	0.907
Lysine	1.21	1.27	1.26	1.23	1.25	1.24	1.20	1.27	0.033	0.765
Methionine	0.42	0.44	0.44	0.43	0.43	0.44	0.42	0.44	0.012	0.917
Phenylalanine	0.60	0.61	0.62	0.61	0.61	0.61	0.60	0.61	0.016	0.989
Threonine	0.64	0.66	0.67	0.66	0.66	0.66	0.64	0.66	0.017	0.952
Tryptophan	0.13	0.13	0.13	0.13	0.12	0.13	0.13	0.13	0.004	0.841
Valine	0.74	0.76	0.76	0.75	0.76	0.76	0.74	0.77	0.033	0.967
Nonessential AAs										
Alanine	1.08	1.06	1.10	1.10	1.04	1.13	1.08	1.07	0.033	0.694
Aspartic acid	1.40	1.45	1.46	1.43	1.43	1.44	1.40	1.44	0.037	0.924
Cysteine	0.14	0.15	0.15	0.15	0.15	0.15	0.14	0.15	0.005	0.977
Glutamic acid	2.07	2.14	2.17	2.12	2.10	2.13	2.09	2.14	0.054	0.936
Glycine	1.46	1.37	1.47	1.51	1.33	1.56	1.47	1.39	0.075	0.398
Hydroxylysine	0.05	0.04	0.05	0.05	0.04	0.05	0.05	0.05	0.003	0.209
Hydroxyproline	0.33	0.29	0.31	0.33	0.28	0.36	0.34	0.28	0.034	0.654
Proline	0.81	0.76	0.81	0.83	0.75	0.87	0.81	0.78	0.036	0.376
Serine	0.56	0.57	0.59	0.58	0.57	0.57	0.56	0.56	0.014	0.859
Taurine	0.37	0.37	0.39	0.38	0.38	0.38	0.36	0.38	0.011	0.752
Tyrosine	0.39	0.42	0.42	0.40	0.42	0.41	0.38	0.41	0.017	0.592
Proximate analysis ^∗^										
Moisture	72.74	73.58	72.81	72.45	73.41	73.51	73.66	72.44	0.578	0.585
Crude protein	16.74	16.26	16.61	16.67	16.35	16.46	16.11	16.89	0.251	0.391
Crude fat	7.14	6.97	7.31	7.48	7.00	6.47	6.86	7.63	0.482	0.764
Crude fiber	0.14	0.11	0.13	0.11	0.11	0.11	0.14	0.11	0.018	0.899
Ash	3.15	2.84	3.06	3.14	3.01	3.32	3.09	3.07	0.092	0.063

*Note*: Values represent the mean of five replicates of each diet. Basal = 0% (2.32%), A005 = 0.05% (2.38%), A01 = 0.10% (2.43%), A02 = 0.20% (2.52%), A03 = 0.30% (2.62%), A04 = 0.40% (2.76%), A05 = 0.50% (2.81%), and A065 = 0.65% (2.94%) supplemented arginine (% dietary arginine).

Abbreviation: PSE, pooled standard error.

^∗^Analyzed at the University of Missouri Agricultural Experiment Station Chemical Laboratories (Columbia, Missouri, USA).

### 3.3. Blood Biochemistry

The serum biochemical parameters of Florida pompano are presented in Table 7. None of the measured indicators were significantly affected by dietary treatments at the conclusion of the 12‐week feeding period (*p* > 0.05). Albumin (*p* = 0.566), ALT (*p* = 0.766), AST (*p* = 0.787), cholesterol (*p* = 0.712), glucose (*p* = 0.901), lactate (*p* = 0.827), lactate dehydrogenase (*p* = 0.362), lipase (*p* = 0.595), creatine kinase (*p* = 0.443), and urea (*p* = 0.946) did not noticeable differ among treatments. Similarly, mineral concentrations (calcium, magnesium, phosphate, iron, zinc, sodium, potassium, and chloride) did not differ across dietary Arg levels. Hematocrit values also showed no significant variation among treatments (*p* = 0.446).

**Table 7:** Blood biochemistry parameters of Florida pompano cultured in a clear water recirculating system for 12 weeks fed different arginine levels, stocked at 12 fish tank^−1^ (~44 fish m^−2^) with an initial weight of 11.06 ± 0.27 g (mean ± standard deviation).

Parameters ^∗^	Basal	A005	A01	A02	A03	A04	A05	A065	PSE	*p*‐Value
Albumin (g L^−1^)	13.05	12.92	18.39	12.85	12.90	12.26	12.08	12.88	2.23	0.566
Alanine aminotransferase (ALT) (U L^−1^)	5.20	3.60	4.20	5.20	5.60	5.00	4.80	5.80	0.95	0.766
Aspartate aminotransferase (AST) (U L^−1^)	30.80	35.00	26.00	38.40	36.60	39.20	32.40	35.00	5.83	0.787
Calcium (mmol L^−1^)	3.79	3.59	3.60	3.58	3.65	3.55	3.67	3.73	0.12	0.860
Cholesterol (mmol L^−1^)	4.31	4.08	4.03	4.21	3.91	4.23	4.02	4.14	0.16	0.712
Creatine kinase (U L^−1^)	2556.60	3146.00	1113.80	2167.80	3432.20	2979.40	2542.80	1850.40	749.48	0.443
Glucose (mmol L^−1^)	6.08	5.26	5.54	5.14	5.26	6.12	6.02	6.90	0.96	0.901
Iron (μmol L^−1^)	12.50	11.00	10.20	10.80	11.00	11.40	11.60	11.00	0.76	0.671
Lactate (mmol L^−1^)	9.91	9.30	9.39	7.89	8.09	9.36	8.61	7.19	1.30	0.827
Lactate dehydrogenase (U L^−1^)	122.20	204.40	109.80	145.00	201.60	190.80	161.80	120.60	35.89	0.362
Lipase (U L^−1^)	5.20	5.29	6.36	5.34	5.20	5.51	6.86	5.20	0.71	0.595
Magnesium (mmol L^−1^)	1.81	1.75	1.87	1.76	1.73	1.79	1.75	1.79	0.08	0.955
Phosphate (mmol L^−1^)	5.27	4.94	4.92	5.19	4.84	4.72	4.94	4.64	0.25	0.647
Zinc (μmol L^−1^)	219.10	207.12	193.16	206.62	200.46	198.26	195.08	214.62	10.28	0.586
Sodium (mmol L^−1^)	192.00	170.40	185.40	217.80	198.00	172.00	186.40	172.00	23.57	0.850
Potassium (mmol L^−1^)	7.25	7.44	7.66	6.95	7.67	8.22	8.18	7.50	0.95	0.981
Chloride (mmol L^−1^)	140.00	143.00	142.80	150.40	140.20	143.40	140.60	142.20	3.51	0.515
Urea (mmol L^−1^)	0.33	0.34	0.28	0.30	0.30	0.32	0.30	0.28	0.04	0.946
Hematocrit (%)	48.07	49.13	46.07	49.33	45.60	45.40	42.87	44.20	2.32	0.446

*Note*: Values represent the mean of five replicates of each diet. Basal = 0% (2.32%), A005 = 0.05% (2.38%), A01 = 0.10% (2.43%), A02 = 0.20% (2.52%), A03 = 0.30% (2.62%), A04 = 0.40% (2.76%), A05 = 0.50% (2.81%), and A065 = 0.65% (2.94%) supplemented arginine (% dietary arginine).

Abbreviation: PSE, pooled standard error.

^∗^Analyzed by WellFish Tech (Charlottetown, Prince Edward Island, Canada).

### 3.4. Gene Expression

Relative expression of hepatic and intestinal target genes is presented in Figures 2 and 3, respectively. Dietary Arg supplementation had no significant effect on the expression of any target gene in either tissue (*p* > 0.05). In the liver (Figure 2), the expression of peroxisome proliferator‐activated receptor alpha (*ppara*; *p* = 0.945), insulin‐like growth factor 1 (*igf1*; *p* = 0.642), target of rapamycin (*tor*; *p* = 0.722), and manganese superoxide dismutase (*mnsod*; *p* = 0.806) did not differ among treatments, with fold‐change values remaining similar across all levels. In the distal intestine (Figure 3), expression of occludin (*ocln*; *p* = 0.329), zonula occludens‐1 (*zo1*; *p* = 0.421), lysozyme (*lyz*; *p* = 0.722), and interleukin‐8 (*il8*; *p* = 0.760) likewise did not differ among treatments, with fold‐change values fluctuating around 1.0 regardless of Arg inclusion.

**Figure 2:**
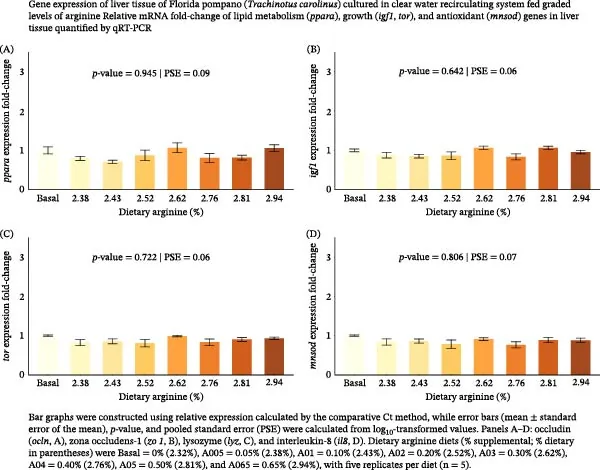
Relative expression of hepatic target genes in juvenile Florida pompano (*Trachinotus carolinus*) fed graded dietary arginine levels for 12 weeks. Panels show expression of (A) *ppara*, (B) *igf1*, (C) *tor*, and (D) *mnsod*.

**Figure 3:**
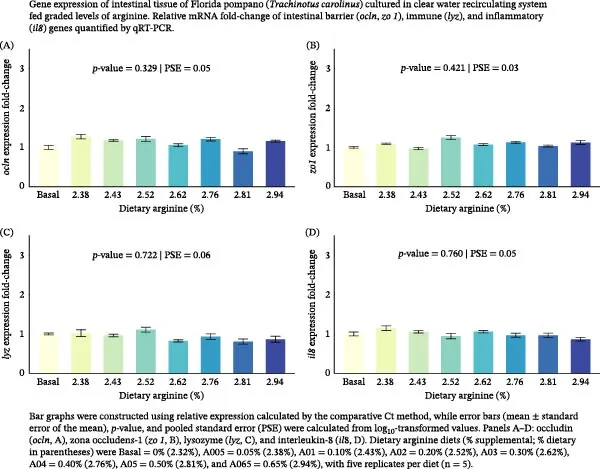
Relative expression of intestinal target genes in juvenile Florida pompano (*Trachinotus carolinus*) fed graded dietary arginine levels for 12 weeks. Panels show expression of (A) *ocln*, (B) *zo1*, (C) *lyz*, and (D) *il8*.

## 4. Discussion

The present study investigated the effects of dietary Arg supplementation on juvenile Florida pompano (*Trachinotus carolinus*) through a dose–response analysis across eight dietary Arg concentrations ranging from 2.32% to 2.94% of the diet (as‐is basis). A practical low animal protein diet in combination with a 4% crystalline AA premix was used to reduce the Arg level to 5.66% of dietary protein, a level below the reported dietary requirement of 6.32%–6.35% of dietary protein for golden pompano [16]. Contrary to initial expectations, growth performance parameters, including FW, WG, TGC, and FCR, showed no significant differences among dietary treatments, suggesting that the basal diet with an Arg concentration of 2.32% (5.66% of dietary protein) was adequate to support growth performance under the experimental conditions. While the dietary Arg gradient was created using crystalline L‐arginine supplementation, all diets shared a common intact protein matrix in which poultry meal, SBM, and corn protein concentrate contributed the majority of basal dietary Arg. This differs from fully purified diet requirement studies in which protein‐bound and free AA pools are absent or minimized, and absorption of predominantly intact protein Arg may result in a bioavailable pool not fully captured by total analyzed dietary concentrations alone [76]. Along with certain other AAs, Arg is currently recognized as a potential conditionally EAA, with endogenous synthesis through citrulline and proline interconversion sparing dietary Arg [77]. At the juvenile growth stage, maintenance accounts for roughly 10% or less of the requirement, so Arg demand is dominated by growth and is met from intact‐protein Arg plus endogenous synthesis [78]. The apparent Arg requirement for Florida pompano therefore appears to be ≤2.32%, a value lower than those reported for golden pompano, which requires 2.73%–2.74% of diet (6.32–6.35% of dietary protein) [16], cobia (*Rachycentron canadum*) 2.82% of diet (6.13% of dietary protein) [79], and red sea bream (*Pagrus major*) 2.37% of the diet (4.74% of dietary protein) [80]. The basal dietary Arg level of 2.32% in the present study exceeded the reported requirements for red drum (*Sciaenops ocellatus*) 1.75% of diet (5% of dietary protein) [31, 81] and Japanese flounder (*Paralichthys olivaceus*) 2.04% of diet (4.08% of dietary protein) [82], suggesting that the Arg requirement of Florida pompano may fall below 2.32% and potentially within the range observed for these species, as well as European sea bass (*Dicentrarchus labrax*) and Asian sea bass (*Lates calcarifer*), where growth reached a plateau at ~1.8% dietary Arg (3.8%–3.9% of dietary protein) [83, 84]. The lower Arg requirement of Florida pompano relative to golden pompano may reflect species differences in endogenous Arg synthesis, including a potentially greater capacity for Arg‐sparing through citrulline and proline interconversion [77], though this hypothesis requires direct investigation in *T*. *carolinus* through isotopic tracing or tissue citrulline quantification.

While the overall growth data indicate no significant treatment effects, a secondary numerical pattern warrants brief consideration. A numerical trend indicated slightly higher WG in fish fed intermediate Arg levels (2.52%–2.76% of diet) than in fish fed the basal and highest supplementation levels. While not statistically significant, this pattern resembles the quadratic response often observed in AA dose–response studies, where performance increases with supplementation up to a threshold concentration and then plateaus or declines at excessive levels [85]. The slight decline at the highest Arg concentrations (2.81%–2.94% of diet) could theoretically reflect AA imbalances, particularly antagonism between Arg and lysine. Both compete for common cationic AA transporters (CAT‐1 and CAT‐2) at the intestinal brush border membrane [76, 86], and excess dietary Arg has been shown to impair lysine absorption and utilization in terrestrial animals [87]. Similar antagonistic relationships have been reported in fish including channel catfish (*Ictalurus punctatus*) [88], rainbow trout (*Oncorhynchus mykiss*) [89, 90], Atlantic salmon (*Salmo salar* L.) [91], and black sea bream (*Acanthopagrus schlegelii*) [92], although direct evidence in marine species remains limited and no direct measurement of intestinal transporter activity, plasma lysine, or whole‐body lysine content was conducted in the present study, so the antagonism hypothesis remains speculative. Beyond competitive inhibition at shared intestinal transporters, excess dietary Arg may increase hepatic arginase activity, converting Arg to ornithine and urea and potentially reducing the availability of other AAs for protein synthesis [93].

The FCR values across all treatments indicate high feed utilization efficiency consistent with optimized practical diets for pompano [7, 8, 94]. The lack of significant differences in FCR among treatments aligns with the growth data and further supports the conclusion that the basal Arg level was adequate. Likewise, ANPR values remained stable across all Arg concentrations, suggesting that protein synthesis was not limited by Arg availability within the tested range. These values align with those reported for yellow grouper (*Epinephelus awoara*) [95], golden pompano [16], and red drum (*Sciaenops ocellatus*) [81] in similar studies. The stability of ANPR indicates that nitrogen utilization efficiency was not compromised by either Arg deficiency or AA imbalance despite Arg’s critical role in the urea cycle and NO synthesis [96].

Whole‐body proximate and AA profiles remained unchanged among treatments, indicating that dietary Arg levels within the tested range did not alter tissue composition or protein quality. This aligns with findings in other species in which AA requirements are typically defined by growth response rather than carcass composition as the latter is more conserved [16, 24, 95, 97]. The consistency in whole‐body AA profiles across dietary Arg concentrations suggests effective homeostatic regulation of tissue AA composition, likely mediated by adjustments in AA oxidation and protein turnover [98]. Even the highest Arg supplementation did not elevate whole‐body Arg content, suggesting efficient catabolism of excess Arg via arginase, which converts Arg into ornithine and urea [93].

Serum biochemical parameters and mineral concentrations remained within normal physiological ranges, with stable liver enzyme activities (alanine aminotransferase (ALT) and aspartate aminotransferase (AST)), indicating that hepatic function was not compromised by Arg either deficiency or excess. Elevated ALT and AST are typically indicative of hepatocellular damage or metabolic dysfunction [99], and neither enzyme was elevated across any dietary treatment, indicating that liver health was not compromised within the tested Arg range. Serum urea levels, which reflect nitrogen metabolism and can be influenced by AA catabolism, remained stable across all Arg concentrations, consistent with the ANPR data and suggesting that dietary Arg levels did not substantially alter the whole‐body nitrogen balance. In mammals, excessive Arg intake can increase urea production via enhanced arginase activity [93], but the present results suggest that Florida pompano effectively regulated nitrogen metabolism within the tested Arg range. Consistent hematocrit values further suggest that red blood cell volume was not affected by dietary Arg level, indicating no gross disruption of oxygen‐carrying capacity. Since Arg serves as a precursor for NO, which regulates vascular tone and blood flow [100], Arg deficiency could theoretically impair cardiovascular oxygen delivery [101, 102], but the stability of hematocrit across all treatments suggests that this threshold was not approached within the tested Arg range.

The gene expression profiles in both hepatic and intestinal tissues were consistent with the growth and physiological data, providing molecular‐level support for the conclusion that basal dietary Arg was adequate under the experimental conditions. In the liver, uniform *ppara* expression across all dietary treatments suggests that hepatic fatty acid catabolism was not detectably modulated by Arg availability. In other fish species where dietary Arg imposed a physiologically meaningful and sufficiently wide dietary gradient, *ppara* and downstream fatty acid β‐oxidation genes have been shown to respond. In juvenile Atlantic salmon, higher dietary Arg stimulated hepatic fatty acid β‐oxidation through increased carnitine palmitoyltransferase‐1 (CPT‐1) expression, an effect linked to greater polyamine turnover and reduced acetyl‐CoA availability [101, 103]. Similarly, Arg supplementation reduced liver lipid accumulation by upregulating *ppara* and related fatty acid oxidation genes in Nile tilapia (*Oreochromis niloticus*) fed high‐fat diets [104]. In both cases, the *ppara* response occurred under conditions of a wide Arg range or a high‐fat metabolic challenge that placed measurable demand on hepatic fat oxidation. In the present study, the 8% dietary lipid level and the predominantly intact protein Arg background likely provided insufficient stimulus to produce a detectable shift in *ppara* expression, and no comparable *ppara* modulation was detected.

The stable expression of *igf1* and *tor* across all Arg concentrations is consistent with the growth data. In fish species where dietary Arg was limiting, both genes have been shown to respond. In gibel carp (*Carassius auratus gibelio*), *tor* and *igf1* expression in liver and muscle were significantly downregulated in fish fed Arg‐deficient diets, while plasma *igf1* and growth hormone concentrations increased with Arg supplementation toward the requirement level [105]. Juvenile blunt snout bream (*Megalobrama amblycephala*) showed a similar pattern, with *igf1* and *tor* expression peaking at optimal Arg and declining below it [106], consistent with the established role of Arg in activating *tor* signaling to support protein synthesis in fish [78]. Considering that neither gene was downregulated at the lowest tested Arg concentration in the present study confirms that basal dietary Arg was sufficient to sustain normal anabolic signaling in Florida pompano. For *mnsod*, expression has been shown to respond to dietary Arg through the Nrf2/Keap1 pathway in fish intestine, where activity and gene expression are highest at optimal Arg and reduced at both deficient and excessive concentrations [107]. A similar pattern in hepatic antioxidant gene regulation has been reported in other species, though direct evidence in liver tissue remains limited [108]. The lack of *mnsod* modulation, combined with stable serum ALT and AST activities, suggests that the hepatic oxidative status was not affected by dietary Arg within the tested range.

In the distal intestine, stable *ocln* and *zo1* expression across all dietary treatments indicates that tight junction organization and paracellular barrier integrity were maintained regardless of dietary Arg concentration. Tight junction gene expression in fish responds to Arg availability at both ends of the dose–response curve. In Jian carp (*Cyprinus carpio* var. Jian), Arg deficiency upregulated occludin and claudin 7 mRNA as a likely compensatory stress response, while antioxidant enzyme activity and immune gene expression were concurrently impaired [109]. In grass carp (*Ctenopharyngodon idellus*) enterocytes, *ocln* and *zo1* expression peaked at sufficient Arg and declined with excess through TOR signaling [110]. Neither pattern appeared in Florida pompano, suggesting that the tested Arg range did not stress or overstimulate the intestinal epithelium. Mucosal immune status followed the same trend, with *lyz* and *il8* expression unaffected across all treatments. In Senegalese sole (*Solea senegalensis*) subjected to repeated handling stress, dietary Arg supplementation enhanced *lyz* expression and disease resistance against *Photobacterium damselae* subsp. *piscicida* in a dose‐dependent manner [33]. In golden pompano, dietary Arg at a level not necessary for growth led to maximize lysozyme activity and resistance against *Vibrio harveyi* [16]. Florida pompano showed no such separation under the present conditions, which likely reflects that basal Arg at 2.32% of the diet was sufficient to maintain normal mucosal immune function in unstressed fish, though whether a pathogen or crowding challenge would change that cannot be confirmed without a controlled challenge study.

Florida pompano can achieve growth with dietary Arg concentrations at or below 2.32% when fed a diet containing 40% crude protein and a balanced AA profile. This finding has practical economic value as it suggests that crystalline Arg supplementation beyond basal levels provided by conventional intact protein sources may be unnecessary under standard production conditions. However, this conclusion comes with important caveats. AA requirements vary with life stage, fish size, water temperature, and physiological state. As this study was conducted with juveniles under controlled temperature conditions, the requirement estimates may not apply to larval stages, broodstock, or fish experiencing thermal stress or crowding. Furthermore, the requirement estimated in this study was based primarily on growth performance as gene expression of mucosal immune markers showed no significant response under the unstressed conditions of the trial. Arg requirements for immune function and stress resistance can exceed those for growth in other species [17, 101], and given that Lin et al. [16] demonstrated in golden pompano that the Arg level supporting peak lysozyme activity and disease resistance against *Vibrio harveyi* exceeded the level required for growth, future work with Florida pompano should establish whether immune function similarly requires higher dietary Arg than what growth alone indicates. Controlled challenge trials using pathogen exposure or crowding stress alongside graded Arg diets, with outcome measures including survival, bacterial tissue loads, head kidney immune gene expression, and plasma NO metabolites, would provide a more complete and production‐relevant Arg requirement estimate for this species.

## 5. Conclusion

This study demonstrates that the dietary Arg requirement of juvenile Florida pompano is met at ≤2.32% of diet (≤5.66% of dietary protein), one of the lower values reported for marine fish. The lack of a growth response to Arg supplementation, coupled with stable feed efficiency, protein retention, serum biochemistry, and gene expression profiles across hepatic and intestinal tissues, indicates that the conventional protein ingredients utilized in this study supply adequate Arg to support normal growth, physiological function, and molecular homeostasis in juvenile Florida pompano. These findings suggest that Arg supplementation may not be required even in low‐animal protein diets, supporting more cost‐effective feed formulation. As immune function or stress resistance is influenced by Arg levels, future studies should include health metrics.

## Author Contributions

**Trinh H. V. Ngo**: data curation, visualization, writing – original draft. **Khanh Q. Nguyen**: writing – review & editing. **Timothy J. Bruce**: methodology, writing – review & editing. **Nicholas Romano**: writing – review & editing. **Marty Riche**: writing – review & editing. **D. Allen Davis**: conceptualization, methodology, writing – review & editing, funding acquisition, supervision, project administration.

## Funding

This research was supported in part by the Hatch Funding Program (Grant ALA016‐1‐19102) of the Alabama Agricultural Experiment Station. This research was supported in part by the U.S. Department of Agriculture, Agricultural Research Service, by a cooperative agreement (Number 59‐6034‐9‐007) with Florida Atlantic University under the project number 6034‐22000‐047‐000D.

## Disclosure

All the authors reviewed and approved the manuscript. The authors confirm that they conducted all research and prepared the manuscript without the involvement of additional persons, third‐party services, or artificial intelligence software.

## Ethics Statement

All experimental procedures involving the care and use of fish in this study were reviewed and approved by the Institutional Animal Care and Use Committee (IACUC) at Auburn University under protocol number 2021‐3989.

## Conflicts of Interest

The authors declare no conflicts of interest.

## Data Availability

Data are available upon request from the authors.
